# New insight into biodegradable macropore filler on tuning mechanical properties and bone tissue ingrowth in sparingly dissolvable bioceramic scaffolds

**DOI:** 10.1016/j.mtbio.2023.100936

**Published:** 2023-12-28

**Authors:** Xiaoyi Jiao, Fanghui Wu, Xusong Yue, Jun Yang, Yan Zhang, Jiandi Qiu, Xiurong Ke, Xiaoliang Sun, Liben Zhao, Chuchu Xu, Yifan Li, Xianyan Yang, Guojing Yang, Zhongru Gou, Lei Zhang

**Affiliations:** a Department of Orthopaedic Surgery, The First Affiliated Hospital of Wenzhou Medical University, Wenzhou, 325000, China; b Department of Orthopaedics, The Third Hospital Affiliated to Wenzhou Medical University & Rui'an People's Hospital, Rui'an, 325200, China; c Bio-nanomaterials and Regenerative Medicine Research Division, Zhejiang-California International Nanosystem Institute, Zhejiang University, Hangzhou, 310058, China; d Zhejiang Provincial Clinical Research Center for Oral Diseases, Key Laboratory of Oral Biomedical Research of Zhejiang Province, Cancer Center of Zhejiang University, Stomatology Hospital, School of Stomatology, Zhejiang University School of Medicine, Hangzhou, 310006, China; e Department of Orthopaedics, The First Affiliated Hospital, School of Medicine of Zhejiang University, Hangzhou, 310003, China

**Keywords:** Guided bone regeneration, 3D printing, Wollastonite bioceramics, Hardystonite, Controlled biodegradation

## Abstract

Structural parameters of the implants such as shape, size, and porosity of the pores have been extensively investigated to promote bone tissue repair, however, it is unknown how the pore interconnectivity affects the bone growth behaviors in the scaffolds. Herein we systematically evaluated the effect of biodegradable bioceramics as a secondary phase filler in the macroporous networks on the mechanical and osteogenic behaviors in sparingly dissolvable bioceramic scaffolds. The pure hardystonite (HT) scaffolds with ∼550 & 800 μm in pore sizes were prepared by digital light processing, and then the Sr-doped calcium silicate (SrCSi) bioceramic slurry without and with 30 % organic porogens were intruded into the HT scaffolds with 800 μm pore size and sintered at 1150 °C. It indicated that the organic porogens could endow spherical micropores in the SrCSi filler, and the invasion of the SrCSi component could not only significantly enhance the compressive strength and modulus of the HT-based scaffolds, but also induce osteogenic differentiation of bone marrow mesenchymal stem cells (BMSCs). The pure HT scaffolds showed extremely slow bio-dissolution in Tris buffer after immersion for 8 weeks (∼1 % mass decay); in contrast, the SrCSi filler would readily dissolve into the aqueous medium and produced a steady mass decay (>6 % mass loss). *In vivo* experiments in rabbit femoral bone defect models showed that the pure HT scaffolds showed bone tissue ingrowth but the bone growth was impeded in the SrCSi-intruded scaffolds within 4 weeks; however, the group with higher porosity of SrCSi filler showed appreciable osteogenesis after 8 weeks of implantation and the whole scaffold was uniformly covered by new bone tissues after 16 weeks. These findings provide some new insights that the pore interconnectivity is not inevitable to impede bone ingrowth with the prolongation of implantation time, and such a highly biodegradable and bioactive filler intrusion strategy may be beneficial for optimizing the performances of scaffolds in bone regenerative medicine applications.

## Highlights

• HT-based bioceramic scaffold with dynamically changing pore structures was prepared for bone defect repair.
• Sr-doped CSi filler significantly enhanced the bioactivity and osteoinductivity of the HT scaffold.
• Sr-doped CSi filler exhibited rapid biodegradation and bioactive ion release.
• The group with microporous SrCSi filler showed excellent osteogenic capacity and new bone ingrowth.

## Graphical abstract

**Figure:**
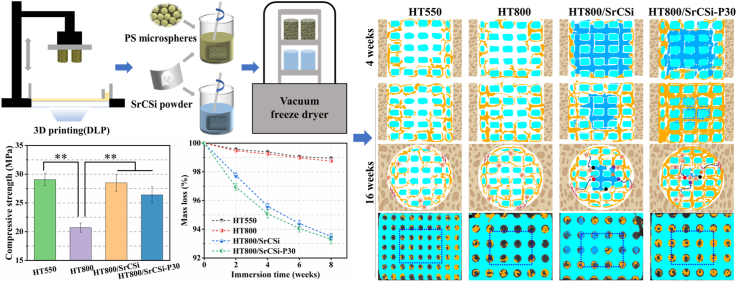

## 1 Introduction

Bone is a highly vascularized and fully regenerative tissue with exceptional self-repairing ability. Minor fractures or defects typically heal on their own without the need for specific treatments [1]. However, when bone defects exceed the critical size due to various reasons, healing may be delayed or completely hindered since they require extensive bone regeneration and lack the necessary structural support. In such cases, surgical intervention is indicated [2,3]. Currently, a variety of artificial grafts, such as tantalum, titanium alloys, and polyether ether ketone (PEEK), are widely available for reconstructing and repairing substantial bone defects [[4], [5], [6]]. These metallic scaffold implants exhibit robust mechanical properties, making them attractive options for treating large bone defects [7]. However, due to their limited bioactivity, they face challenges in promoting substantial bone regeneration and ingrowth. To address this limitation, many studies have explored surface modifications, but these processes can be exceedingly intricate [8,9]. Additionally, non-degradable scaffolds can lead to chronic inflammation and may necessitate secondary surgeries [10]. More recently, biodegradable polymers like polylactic acid, chitosan, and citrate have attracted significant interest. While these polymers share similar organic compositions with natural tissue, their mechanical properties and osteogenic capacity are often insufficient for clinical applications [11,12]. It is widely recognized that ideal implants must strike a balance between mechanical strength and osteogenic capacity while gradually bio-resorbing after regenerating damaged bone tissue.

Bioceramic scaffolds have been recognized as a group of promising candidates for promoting bone repair due to their favorable physicochemical and biological properties [[13], [14], [15]], whereas some clinical bone repair results filled with bioceramic scaffolds are often unsatisfactory. To address this, it is reasonable to postulate that optimizing pore geometry in bioceramic scaffolds may be a promising strategy to enhance osteoconductive performance. In fact, scaffold geometries such as pore shape, size, and interconnectivity can control cell proliferation and aggregation, thereby tuning the regeneration efficiency of bone tissues [[16], [17], [18]]. Karageorgiou et al. reported that the pore size determines the regeneration pathway of bone, with smaller pore sizes leading to hypoxia and cartilage formation and larger pore sizes leading to direct bone formation, and that bone tissue regeneration improves with increasing pore size [16,19]. However, the large pore size may be detrimental to the structural integrity of the scaffold, meaning that it does not provide adequate mechanical support at load-bearing sites. In addition, larger pore size may lead to peripheral soft tissue invasion and poor bone healing, especially in bone defects in sites lacking periosteal protection [20]. Therefore, the development of bioceramic implants that integrate structure and function for the repair of complex bone defects remains a formidable challenge. The in-situ regenerative response triggered by bone implants plays a pivotal role in tissue reconstruction [21]. It is now understood that the key lies in modulating the local immune microenvironment and osteogenesis-related genes to enhance osteogenic properties [22]. Rapid vascularization and infection prevention are also vital factors for the success of implants. In this context, hardystonite (HT; Ca_2_ZnSi_2_O_7_) bioceramics have become research hotspots due to their favorable biocompatibility and potential antibacterial properties [23,24]. Wu et al. demonstrated that HT bioceramics may promote the value-addition and differentiation of osteoblasts [25]. Although this bioceramic is suboptimal for bone regeneration and repair due to its limited *in vivo* biodegradability [26], the inorganic ions released during its surface biodegradation yield a synergistic effect. These ions not only promote endothelial value addition and blood vessel growth but also effectively inhibit bacterial colonization on the ceramic material's surface [27,28]. Therefore, it would be beneficial to optimize the pore modification of HT bioceramic scaffolds to promote bone tissue ingrowth and reconstruct challenging bone defects.

Over the years, several other calcium-silicate ceramics have been widely studied for use as bone implants. For example, wollastonite (CaSiO_3_; CSi) is considered a promising material for bone repair due to its favorable biocompatibility and bioactivity [29]. The release of Ca and Si ions from CSi during its degradation can enhance both vascularization and bone regeneration *in vivo*. Additionally, it can stimulate biomimetic hydroxyapatite (HA) re-mineralization and deposition, expediting osseointegration [30]. Unfortunately, the fast biodegradation rate and mechanical decay of pure CSi bioceramics limit its applications [31]. Consequently, the structural reliability issue of CSi bioceramic can limit the advancement of such porous CSi bioceramic scaffolds. However, few studies have been reported on the use of CSi ceramics as secondary relative ceramic scaffolds for filler modification to improve the mechanical properties of the scaffolds and induce bone tissue regeneration by taking advantage of their high bioactivity and rapid degradation properties.

In this context, herein we expand the conventional fully pore interconnectivity strategy to develop another group of HT-based bioceramic composite scaffolds with hidden pore architectures by filling highly biodegradable secondary component of CSi to mediate their mechanical properties and osteogenic capability with time *in vivo*. The macroporous HT scaffolds with complete pore interconnectivity were firstly prepared by digital light processing (DLP) process and then the CSi doped with strontium were intruded into the macropore networks of HT scaffolds. Sr, a divalent cation and essential trace element in bone metabolism, is used for the treatment of osteoporosis and increased bone density [32]. It has been reported that doping Sr into chalcogenide bioceramics not only accelerates their biodegradation but also improves the osteogenic properties of chalcogenide ceramics [33]. In this study, to further understand the influence of the biodegradation rate of SrCSi filler on the bioceramic composite scaffolds, 30 % polystyrene (PS) microspheres were used as porogens in the SrCSi component. The mechanical properties, *in vitro* biolysis, and osteogenic responses of the HT-based bioceramic scaffolds with varying pore sizes and filler microstructures were systematically evaluated.

## 2 Materials and methods

### 2.1 Materials

The inorganic and organic reagents sodium silicate (Na_2_SiO_3_·9H_2_O), calcium nitrate tetrahydrate (Ca(NO_3_)_2_·4H_2_O), strontium nitrate (Sr(NO_3_)_2_), zinc nitrate hexahydrate (Zn(NO_3_)_2_·6H_2_O) and tetraethyl orthosilicate ((C_2_H_5_O)_4_Si, TEOS) for experiments are bought from Sinopharm Reagent Co., Shanghai. The photosensitive resin for printing was supplied by Ten Dimensions Technology Co., China. Trishydroxymethylaminomethane (Tris) was purchased from Aladdin Biological Technology Co., China. All reagents were used directly without further processing.

### 2.2 Preparation and characterization of ceramic powders

The HT powders were synthesized by the sol-gel method, as previously described [34]. Briefly, TEOS, ethanol, and HNO_3_ were mixed in a certain ratio and stirred to hydrolyze for 0.5 h. Then Zn(NO_3_)_2_·6H_2_O and Ca(NO_3_)_2_·4H_2_O were added to the clarified solution after complete hydrolysis and stirred thoroughly for 4 h. The mixed solution was sealed at 60 °C for 36 h to form a gel, which was then dried at 120 °C for 72 h. The dried gel obtained was calcined at 1250 °C for 4 h. The SrCSi powders were synthesized by wet chemical precipitation [35]. All powders were milled for 8 h in a planetary ball mill (Chishun Sci&Tech Co., China) with a certain percentage of ethanol solution as a medium to obtain ultrafine particle-size powders (<5 μm). X-ray diffraction (XRD, Rigaku, Tokyo, Japan) and inductively coupled plasma-optical emission spectroscopy (ICP-OES; Thermo, UK) were used to verify the phase and elemental composition (calcium, strontium, silicon) of the synthesized powders.

### 2.3 Preparation of bioceramic scaffolds

**Scheme 1:**
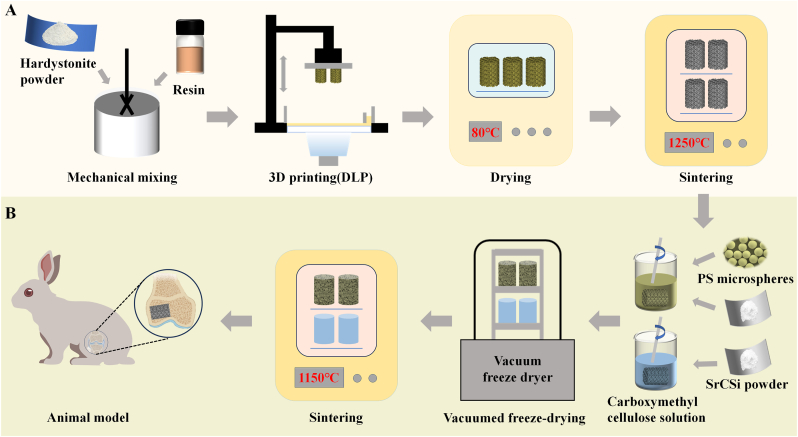
(A) Technology roadmap for pure HT ceramics scaffold preparation. (B) Preparation of biphasic composite ceramic scaffolds and schematic illustration of animal models.

Two porous scaffold models with pore sizes of approximately 550 & 800 μm were designed using Magics software (version 21, Materialise, Leuven, Belgium). The specific methodology and process for the design of this model have been described in our previous studies [36]. The HT powders (60 wt%) were mixed with photosensitive resin and ball-milled for 2 h. The mixture was then poured into a 200-mesh sieve to obtain the printed ceramic slurry. A DLP 3D printer (Ten Dimensions Technology Co., China) was used to print the scaffold samples layer by layer according to the pre-set structural model and printing parameters under 405 nm UV light irradiation. After printing, the green samples were cleaned with deionized water and ethanol and then dried at 80 °C. The dried green samples were kept in a muffle furnace at 1250 °C for 3 h and then cooled with the furnace (Scheme 1A). The two groups of pure HT scaffolds obtained were named HT550 and HT800, respectively.

To obtain the SrCSi-infused scaffolds, 15.0 g of SrCSi powder (the pore-making group was replaced by 30 wt% polystyrene (PS) microspheres with 15 μm diameter) was dispersed in 10 ml of 1.2 % carboxymethyl cellulose solution and mixed thoroughly to obtain the composite slurry. The HT800 scaffold was completely submerged in the slurry and filled the internal pores of the scaffold under vacuum conditions, and the excess slurry was wiped from the surface. After drying, the resulting scaffolds were sintered at 1150 °C for 3 h (Scheme 1B). The sintered biphasic bioceramic scaffolds were denoted as HT800/SrCSi and HT800/SrCSi–P30, respectively.

### 2.4 Primary characterization analysis

The external appearance of the scaffolds was assessed using a digital camera (SONY). The microstructure of the scaffold surface and the SrCSi filler was analyzed by scanning electron microscopy (SEM; Gemini 300, Zeiss, Germany), and the pore size of the scaffolds (pore diameter in the top view) was measured. The scaffolds were evaluated using microcomputed tomography (XM, Hiscan, China) to analyze their internal pore arrangement, mean pore dimensions, specific surface area, and porosity. The built-in device software (IAW, Siemens, Germany) was used to generate 3D reconstructed images and 2D cross-sectional images. The selected sample area was designated as the region of focus (ROF). Subsequently, the aggregate ROF volume, scaffold volume, and overall porosity (defined as porosity = (1 - scaffold volume/ROF volume) × 100 %) were calculated.

### 2.5 *In vitro* mechanical and biodegradable property evaluation

A universal testing machine (Instron 5566, Germany) was used to determine the compressive strength and elastic modulus of bioceramic scaffolds (n = 5). The crosshead velocity was adjusted to 0.5 mm per minute according to the procedure outlined in ASTMC773-88. Four groups of columnar scaffolds (M_0_; *n* = 5) were individually weighed and then immersed in Tris buffer (pH 7.4) at 37 °C, maintaining a solid-liquid ratio of 1.0 g/50 ml. At the specified immersion time interval, 1.0 ml of supernatant liquid was sampled and the concentration of ion release (Ca^2+^, Si^4+^, Zn^2+^, Sr^2+^) was measured using inductively coupled plasma (ICP; Thermo, USA), with an equal volume of new buffer added to the immersion solution. At the corresponding time points, the scaffolds were removed, cleaned, and weighed (M_n_) after their weight had stabilized. The percentage of mass loss was calculated as mass loss = M_n_/M_0_ × 100 %. Subsequently, the compressive strength of the scaffolds after soaking for different times was measured. Furthermore, after a 7-day immersion in simulated body fluid (SBF), SEM-EDS techniques were used to detect the deposition of apatite on the surface of the scaffolds, assessing their biomimetic re-mineralization (*in vitro* bioactivity).

### 2.6 *In vitro* cell experiments and osteogenic differentiation assay

#### 2.6.1 Cell culture

rBMSCs (rat bone marrow mesenchymal stem cell) were extracted from the femoral bone marrow of 2-week-old male rats and placed in αMEM (Gibco BRL, USA) containing 10 % fetal bovine serum and 1 % penicillin-streptomycin. The cells were incubated at 37 °C in a 5 % CO_2_ oven. The medium was changed every two days. Cells of passages 3–5 were used for further experiments. Four groups of sterilized scaffolds were immersed in αMEM culture medium (1g/50 ml) and placed on a 37 °C shaker at 100 rpm. The supernatant was removed after 72 h and filtered through a 0.22 μm filter. 10 % fetal bovine serum and 1 % penicillin-streptomycin were then added for subsequent cell culture.

#### 2.6.2 Cytotoxicity assay

Cell viability was tested using CCK-8 (Biosharp, China). The cells of different groups were all seeded on a 96-well culture plate at a density of 2 × 10^3^ cells/well. Cell viability was determined by incubating the cells for 1, 3, and 7 days in a humidified incubator with 5 % CO_2_ at 37 °C. Next, 10 μL CCK-8 solution was added to each well and incubated for 2 h. Finally, the absorbance value at 450 nm was measured using a microplate reader (Infinite F50; TECAN, Hombrechtikon). Meanwhile, to detect the effect of the scaffold on the viability of rBMSCs, a live/dead staining assay was performed on the cells after 24 h of incubation. The samples were stained using Calcein/PI Live/Dead Viability/Cytotoxicity Assay Kit (C2015 M, Beyotime, China). Cell viability (live cells in green, dead cells in red) was then observed using a fluorescence microscope (IX73, Olympus, Japan).

#### 2.6.3 Western blotting and qRT-PCR analysis

To evaluate the effect of scaffolds with different structures on the osteogenic differentiation of rBMSCs, cells were seeded in scaffold extracts. After 7 days of culture, total protein and RNA were extracted using RIPA Lysis Buffer (Beyotime, China) and AG RNAex Pro Reagent (Accurate Biotechnology, China), according to the manufacturer's instructions. Expression of osteogenic differentiation-related proteins (COL1, OPN, and BMP2) and genes (*Col1α1*, *Spp1*, and *Bmp2*) in rBMSCs was detected by Western blotting and real-time quantitative fluorescence polymerase chain reaction (RT-PCR). Primer sequences for each gene are listed in Table S1.

#### 2.6.4 ALP and ARS staining analysis

rBMSCs were separately seeded in a normal medium, and when the cell concentration reached 80 %, the cells were cultured with an extract medium containing 10 mM glycerophosphate, 50 μg/mL l-ascorbic acid, and 10 nM dexamethasone to induce osteogenic differentiation. After 7 days of culture, cells were stained with a BCIP/NBT alkaline phosphatase color development kit (Beyotime; China). In addition, 21 days after osteogenic induction, cells were fixed in 4 % paraformaldehyde solution and then stained with ARS staining solution (PH 4.2, Solarbio, Beijing; China) to measure mineralization deposition.

#### 2.6.5 Immunofluorescence staining testing

rBMSCs were washed with PBS after 7 days of culture and then fixed with 4 % paraformaldehyde for 20 min. The cells were then incubated with Quick-Block™ blocking buffer (Beyotime, China) for staining. It was incubated with primary antibody (anti-OPN) overnight at 4 °C, followed by incubation with anti-rabbit IgG secondary antibody at room temperature. Finally, nuclei and cytoskeleton were stained with DAPI and FITC Phalloidin (Solarbio, Beijing; China), respectively. OPN expression was observed by confocal microscopy (Olympus FV3000).

### 2.7 Animal model and specimen harvesting

To assess the differences in the efficiency of HT-based scaffolds with different internal structures for bone defect repair *in vivo*, the New Zealand rabbit femoral condyle scaffold implantation model was used. All animal experiments were approved by the Animal Ethics Committee of Zhejiang University (No. ZJU20230322). Prior to surgery, the 32 rabbits (2.8 kg) were individually housed in cages for approximately one to two weeks to acclimate to the environment. To minimize the impact of individual variation, the various group's scaffolds were sequentially paired and implanted on both sides of a single rabbit in a completely sterile environment.

Rabbits were anesthetized with 1.5 mg/kg of 2 % phenobarbital sodium (Merck, Germany) via systemic intravenous injection, and excess hair in the surgical area was shaved. After disinfection, a 3–4 cm incision was made on the lateral condyle of the femur, and soft tissues such as muscle and periosteum were separated to fully expose the femoral condyle. A cylindrical defect of critical size (Ø ∼7 × 8.0 mm) was then created on the femoral condyle by a bone drill at 5000 r/min. During the drilling process, the surgical area was continuously flushed with pre-refrigerated saline to reduce local temperature and clean the surgical area to promote wound recovery. The broken bones inside the defect were carefully removed and the scaffold was implanted. After surgery, the rabbits were injected with 400,000 units of penicillin G (400,000 U) daily for 4 days to prevent postoperative infection. At predetermined times (4, 8, and 16 weeks), the rabbits were euthanized by overdose anesthesia, and femoral specimens were collected for further analysis.

### 2.8 X-ray scanning and μCT reconstruction evaluation

The repair of bone defects and the degradation of scaffolds in rabbits were assessed using an X-ray imaging system (XPERT; KUBTEC, USA) operating at 45 KV and 80 μA (n = 5). To further detect the formation and distribution of new bone, all specimens obtained at different time points were scanned and analyzed using a high-resolution CT scanner (Vivact 100, Scanco Medical, Zurich, Switzerland). The defect area was reconstructed using 3D reconstruction software (MIMICS, Medical 21.0, Materialise, Belgium) to further evaluate osteogenic and material degradation indices, including BV/TV (volume fraction of new bone), Tb⋅N (number of bone trabeculae), and RV/TV (volume fraction of remaining material).

### 2.9 Histological analysis

The collected femoral specimens were immersed in a 4 % paraformaldehyde solution and, after 14 days, were removed and rinsed in tap water for 24 h. All samples were dehydrated in ethanol solutions with successive gradient concentrations (70 %, 80 %, 95 %, 100 %) and cleaned with xylene, then embedded in polymethyl methacrylate (PMMA). After the embedded sample hardened, a hard tissue microtome (310CP, EXAKT, Germany) was used to cut it into slices 300 μm thick along the axis of the implanted scaffold. The sections were polished using a grinder (Exakt-Micro-Grindin System, Leica; Germany) to 100–150 μm. The sections were then stained with H&E and McNeal stain.

Histomorphometric observations of the sections were performed under a light microscope (DMLA, Leica; Germany) at different magnifications. The analysis was also performed by the image analysis software Image Pro Plus 6.0 (Media Cybernetic, USA). Quantitative measurements were conducted to determine the area of new bone (BS) and the total area (TS), and subsequently, the ratio of BS/TS was calculated (*n* = 4). To further characterize the phase transition process of the ceramic scaffolds *in vivo*, the polished sections were subjected to SEM and an energy dispersive spectrometer (EDS; Gemini 300, Zeiss, Germany) for scanning and elemental quantitative analysis.

### 2.10 Statistical analysis

Statistical software (SPSS 21.0, IBM, USA) was used to analyze the data, which were then presented as the mean ± standard deviation (SD). The comparison of quantitative data was conducted using a one-way analysis of variance (ANOVA). P < 0.05 means that the results were statistically significant.

## 3 Results

### 3.1 Primary characterization of bioceramic powders and scaffolds

**Fig. 1:**
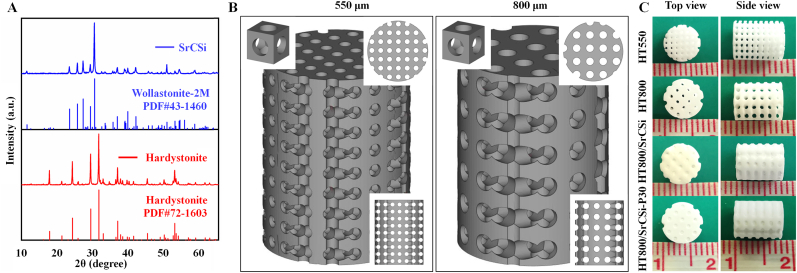
Primary characterization of powder and bioceramic scaffolds. (A) XRD patterns of synthesized SrCSi and HT powders after calcination. (B) Pore geometry of a crystalline cell, CAD, model and its top and side views. (C) Scaffolds after sintering.

XRD analysis confirmed that the synthesized HT and SrCSi powders matched their respective standard peaks for hardystonite (PDF#72-1603) and wollastonite (PDF#43-1460), with no secondary phases (Fig. 1A). Inductively coupled plasma (ICP) analysis revealed that the SrCSi powder contained approximately 4.8 % strontium, closely aligning with the intended theoretical content. To evaluate the influence of SrCSi filler in the cylindrical macropores (∼800 μm) of HT ceramic scaffold, the HT scaffolds (∼550 & ∼800 μm; Ø ∼7 × 8.0 mm) with a theoretical porosity of 50 % were designed by Auto-CAD method (Fig. 1B). The outward appearance of the porous scaffolds after sintering (Fig. 1C) showed that the two groups of pure HT scaffolds maintain the macroporous architectures with full interconnectivity. In contrast, all pore channels of HT800/SrCSi and HT800/SrCSi–P30 group were filled with SrCSi component, and meanwhile, there was no significant deformation and difference in the overall porous structures between the scaffolds.

To prevent substantial shrinkage of the scaffolds during sintering, the 3D-printed structures were scaled up by a factor of 1.2 before printing. Consequently, the actual pore dimensions and porosity of the scaffolds closely matched the intended specifications of the 3D models (Table 1), with samples exhibiting low linear shrinkage (11.4%–12.3 %; P < 0.05). Notably, the secondary sintering at 1150 °C did not affect the morphology of the HT bioceramic scaffolds, but the HT800/SrCSi–P30 group exhibited higher porosity than the HT800/SrCSi group, likely due to the volatilization of organic porogens during the sintering treatment.

**Table 1:** Structural parameters of bioceramic scaffolds.

Sample	Linear shrinkage (%)	Pore size (μm)	Porosity (%)	Specific surface area (1/mm)
HT550	11.4 ± 2.3	545.4 ± 5.1	48.5 ± 2.5	6.47 ± 0.46
HT800	11.8 ± 1.6	797.2 ± 6.6	49.1 ± 1.3	5.10 ± 0.38
HT800/SrCSi	12.3 ± 0.8	–	9.2 ± 1.7	4.11 ± 0.21
HT800/SrCSi–P30	12.1 ± 1.3	–	16.3 ± 1.8	5.21 ± 0.78

### 3.2 Microstructure and bioactivity characterization of scaffolds

**Fig. 2:**
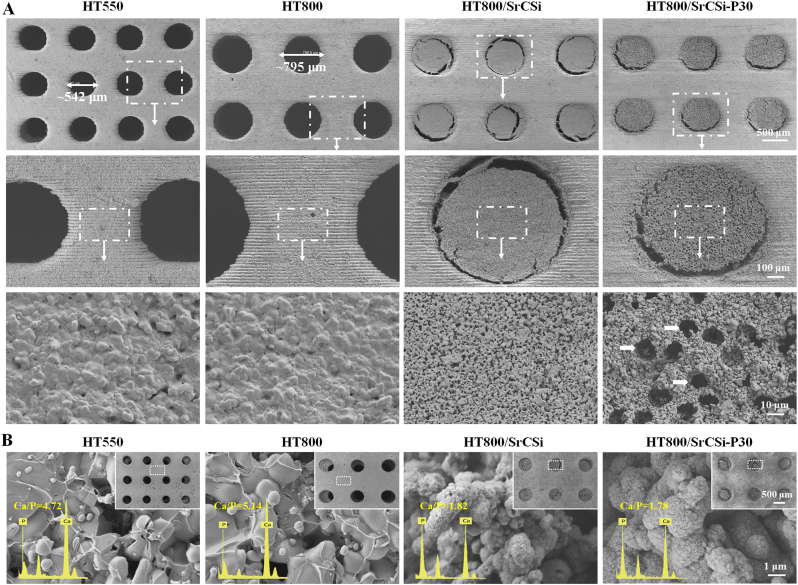
(A) SEM images of scaffolds with different structures and magnified views of labeled areas both showed the structural homogeneity of the scaffolds as expected for composite. White arrows: micropores left after sintering of PS microspheres. (B) Microstructure of apatite coating in locally enlarged area after 1 week of soaking in SBF.

The microstructure and surface characteristics of the bioceramic scaffolds were examined using scanning electron microscopy (SEM) (Fig. 2A). The cylindrical pores were well-preserved in the scaffolds, and the SrCSi filler was observed in the macroporous architecture resulting from the infiltration of the bioceramic slurry. The high-magnification SEM images showed significant differences in sintering and structural densification between the HT and SrCSi bioceramics. In addition, the high-density micropores in the SrCSi filler of the HT800/SrCSi–P30 group could also be observed.

The biomimetic re-mineralization of apatite on the HT scaffold surfaces after one week of immersion in simulated body fluid (SBF) was characterized by SEM-EDX analysis (Fig. 2B). The scaffold geometries remained unaltered after immersion. However, a newly formed surface layer with particle aggregation was observed at high magnification (5000 × ) on the SrCSi-filled HT800 scaffolds, while the pure HT550 and HT800 scaffolds exhibited less surface growth. Quantitative EDX analysis of the Ca/P ratio in the granular surface layer revealed a Ca/P ratio of approximately 1.78–1.82 on the internal SrCSi surface layer, considerably lower than that of the HT surface (4.72–5.14). These results indicate a greater tendency for apatite re-mineralization on the SrCSi surface.

### 3.3 Mechanical and bio-dissolution evaluation of scaffolds

**Fig. 3:**
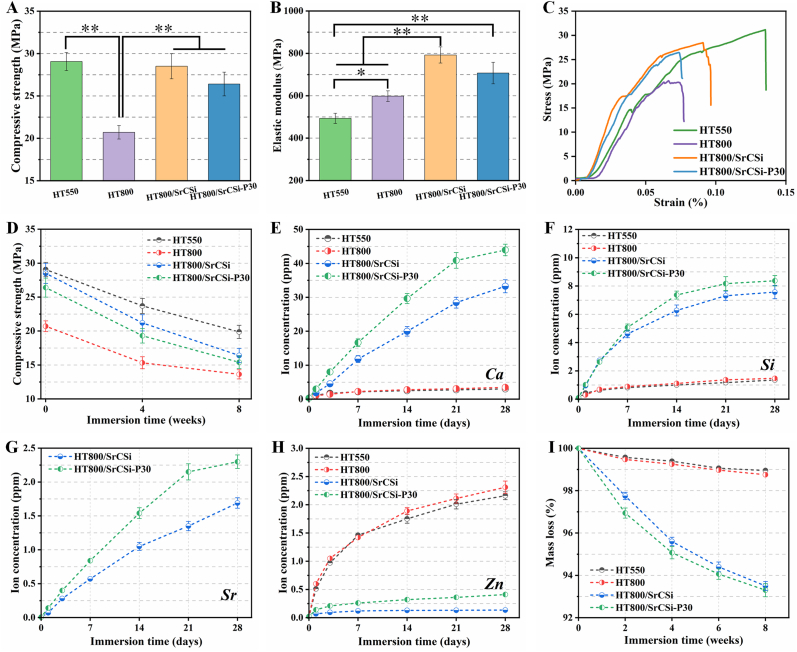
(A, B) Compressive strength and elastic modulus of bioceramic scaffolds. (C) The stress-strain curves of scaffolds. (D) Mechanical decay of bioceramic scaffolds during 8 weeks of immersion. (E–H) Ion release of Ca, Si, Sr, and Zn at corresponding time points in Tris buffer. (I) Mass loss of the stent during 8 weeks of immersion (*p＜0.05, **p＜0.01).

To predict the *in vivo* structural stability of the scaffolds, the scaffold samples were immersed in Tris buffer for an extended period. Before immersion, the compressive strength measurements indicated that while the compressive strength decreased with increasing pore size, all samples exhibited considerable compressive resistance (>20 MPa) (Fig. 3A). The intrusion of the SrCSi component significantly enhanced the compressive strength of the scaffolds, likely due to the reduction of their porosity. However, the HT800/SrCSi–P30 group, with a micropore structure in the SrCSi component, exhibited a less favorable improvement in compressive properties compared to the HT800/SrCSi scaffolds. Moreover, the incorporation of SrCSi notably increased the elastic modulus of the scaffolds (Fig. 3B). Stress-strain curves (Fig. 3C) demonstrated that the scaffolds displayed similar fragmentation characteristics to typical brittle ceramics, with an abrupt decline after a linear increase in stress reached its peak. The compressive strength displayed a gradual decrease with time during immersion (Fig. 3D), particularly with the SrCSi component in the macropores contributing to the compressive resistance compared to the pure HT800 scaffolds.

Ion release and bio-dissolution were observed in all scaffold groups during immersion (Fig. 3E-H), with concentrations of Ca, Si, and Sr ions gradually increasing for the HT800/SrCSi and HT800/SrCSi–P30 groups, while Zn ion concentrations in the pure HT scaffolds increased at a faster rate, attributed to pore closure by the SrCSi component in the composite scaffold groups. Notably, the ionic concentrations of HT800 were similar to those of HT550 at each time point. Furthermore, the mass loss during immersion reflected the substantial bio-dissolution of the SrCSi component in the HT-based scaffolds (Fig. 3I), with the HT800/SrCSi and HT800/SrCSi–P30 groups exhibiting significant mass decay (>5 %), while the pure HT scaffolds exhibited less than 1.3 % mass loss, consistent with the ion release behavior of the bioceramic scaffolds.

### 3.4 Proliferation and osteogenic differentiation of rBMSCs *in vitro*

**Fig. 4:**
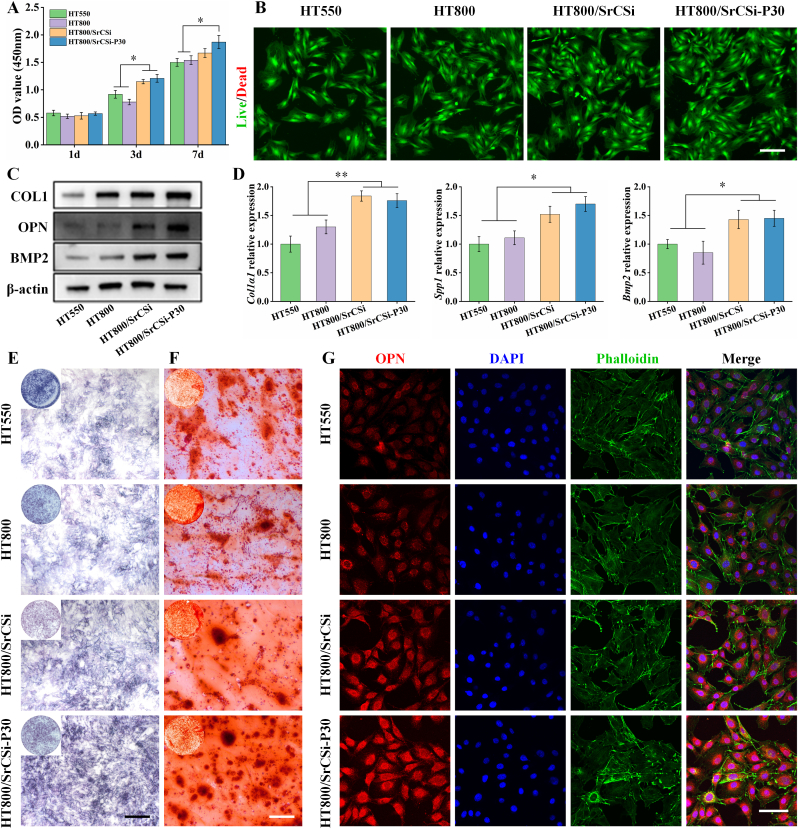
(A) Activity of rBMSCs after 1, 3, and 7 days of culture in different scaffold extracts. (B) Live/dead staining (live cells: green, dead cells: red) of rBMSCs after 24 h of culture. Scale bar = 300 μm. (C) Western blotting images of osteogenesis-related proteins (COL1, OPN, and BMP2) in rBMSCs after treatment with different scaffold extracts. (D) Relative expression of osteogenic genes (*Col1α1*, *Spp1*, and *Bmp2*) in rBMSCs after 7 days of culture in different scaffold extracts. (E) ALP staining of rBMSCs after 7 days of culture in different scaffold extracts. Scale bar = 200 μm. (F) ARS staining of rBMSCs after 21 days of culture in different scaffold extracts. Scale bar = 200 μm. (G) Immunofluorescence staining of OPN in rBMSCs after 7 days of seeding in different extracts. Scale bar = 100 μm. Data represented as mean ± SD (n = 4, *p＜0.05, **p＜0.01). (For interpretation of the references to colour in this figure legend, the reader is referred to the Web version of this article.)

The proliferation of rBMSCs was detected by the CCK-8 kit. The experimental results showed that there was no significant difference in the early stage, and the cell proliferation of both groups of SrCSi-filled scaffolds was significantly enhanced with the extension of culture time (Fig. 4A). The live/dead staining results indicated that most of the cells survived, suggesting good cytocompatibility of the scaffolds ((Fig. 4B). To determine the effect of the scaffolds on the osteogenic differentiation of rBMSCs, the expression of relevant genes and proteins was detected by Western blotting and RT-PCR ((Fig. 4C and D). The results confirmed that the SrCSi-filled scaffolds exhibited higher expression of osteogenic genes *Col1α1*, *Spp1*, and *Bmp2* compared to the pure HT scaffolds. Notably, the concentration of OPN protein bands in the HT800/SrCSi–P30 group was significantly higher than that in the other groups. Alkaline phosphatase staining analysis showed higher ALP expression in both HT800/SrCSi and HT800/SrCSi–P30 groups in comparison with the pure HT scaffolds ((Fig. 4E). Similar results were seen in ARS staining, where the HT800/SrCSi–P30 group exhibited the highest mineralization density ((Fig. 4F). As a key protein regulating bone mineralization and remodeling, immunofluorescence was used to analyze the expression of OPN. It was clear that the addition of the SrCSi component could significantly enhance the expression of OPN ((Fig. 4G).

### 3.5 Preliminary evaluation of bone defect model

**Fig. 5:**
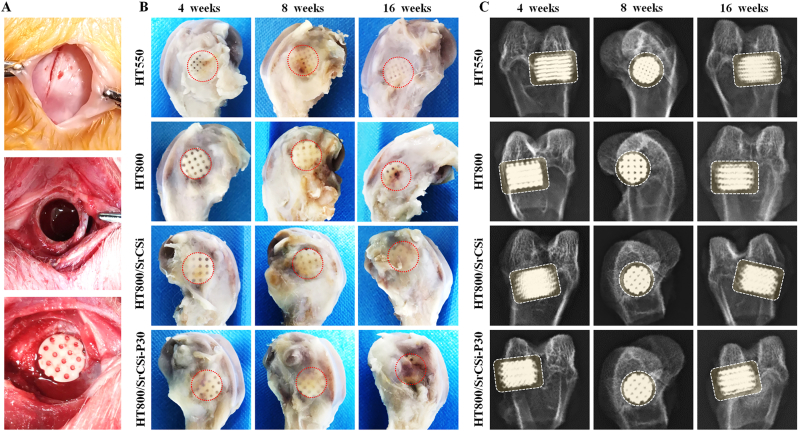
Preliminary evaluation of animal models and experimental results. (A) Preparation of femoral defect model and surgical procedure of scaffold implantation. (B) Femoral specimens at corresponding postoperative time points. (C) X-ray images of femoral specimens 4–16 weeks after ceramic scaffold implantation. Circular or rectangular dashed boxes indicate implanted scaffolds.

The rabbit femoral defect scaffold implantation model was established, as shown in Fig. 5A. Postoperatively, all rabbits did not develop infection and survived to the corresponding time point of specimen collection. Preliminary examination of the femoral specimen showed gradual healing of the bone defect over time without local inflammation or necrosis. At 4 weeks, the newly formed bone scabs partially covered the surface of the implant, and the implanted scaffolds were visible. When the implantation time was extended to 16 weeks, the implanted scaffold was completely covered by well-aligned bone scabs and the implanted scaffolds were indistinguishable, especially in the HT800/SrCSi and HT800/SrCSi–P30 groups (Fig. 5B). The X-ray images showed scaffold biodegradation and new bone formation. The gradual blurring of the material-bone interface in each group at 4–16 weeks postoperatively suggested the expected ingrowth and integration of neo-bone tissue. Notably, the HT800/SrCSi and HT800/SrCSi–P30 groups also showed the original large pore structure left behind by SrCSi degradation (Fig. 5C).

### 3.6 Bone formation measured by μCT

**Fig. 6:**
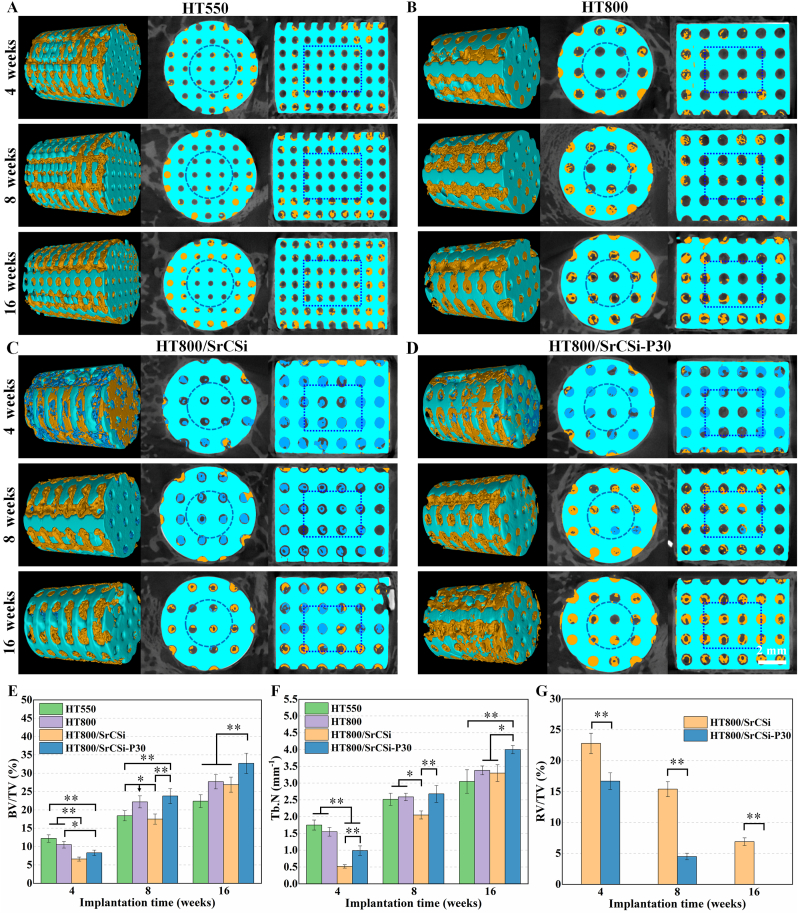
(A–D) μCT reconstruction and cross-sectional images of the bone defect area 4–16 weeks after implantation. Blue: HT scaffold; Green: SrCSi ceramic; Yellow: neo-bone tissue. (E) Quantitative data on BV/TV in areas of bone defects implanted with ceramic scaffolds. (F) The number of neo-bone trabeculae. (G) Quantitative analysis of SrCSi filler degradation throughout the implantation period. Data are expressed as mean ± SD. (*p＜0.05, **p＜0.01). (For interpretation of the references to colour in this figure legend, the reader is referred to the Web version of this article.)

New bone formation at the bone defects at 4–16 weeks postoperatively was visualized through 2D/3D μCT-reconstructed images (Fig. 6A-D). Additionally, BV/TV and Tb**⋅**N were quantitatively measured to compare the osteogenic efficiency of different HT-based scaffolds (Fig. 6E and F). The HT550 and HT800 scaffolds exhibited substantial new bone ingrowth in the peripheral macropores at 4 weeks, with BV/TV exceeding 10 % in both cases. However, limited new bone was present in the SrCSi-filling HT scaffolds, with BV/TV below 8.5 %. At 8 weeks, more neo-bone tissue infiltrated the interior of the HT800 group compared to the HT550, suggesting that the larger pore size was more conducive to sustained new bone ingrowth. It is noteworthy that at this time, the SrCSi filler was gradually degraded inside the biphasic composite ceramic scaffolds and inducing persistent new bone ingrowth. More bone tissue growth was observed in the HT800/SrCSi-p30 group compared to the HT800/SrCSi group, suggesting that the high density of micropores in the former group may be beneficial for increasing the specific surface area and biodegradation of SrCSi filler, thus providing the necessary spatial support for new bone ingrowth. At 16 weeks, the HT800/SrCSi–P30 group displayed the most significant new bone tissue extending into the central region of the HT-based scaffolds, with a BV/TV of 32.7 ± 2.75 %. The neo-bone penetrated the entire porous networks, implying a complete bridging of the neo-bone tissue with the host bone. In contrast, the pure HT550 scaffolds did not exhibit complete bridging by new bone tissue, though it showed inward growth of new bone at an early stage, with a lower BV/TV (<23 %) than the other groups.

### 3.7 Histological analysis

**Fig. 7:**
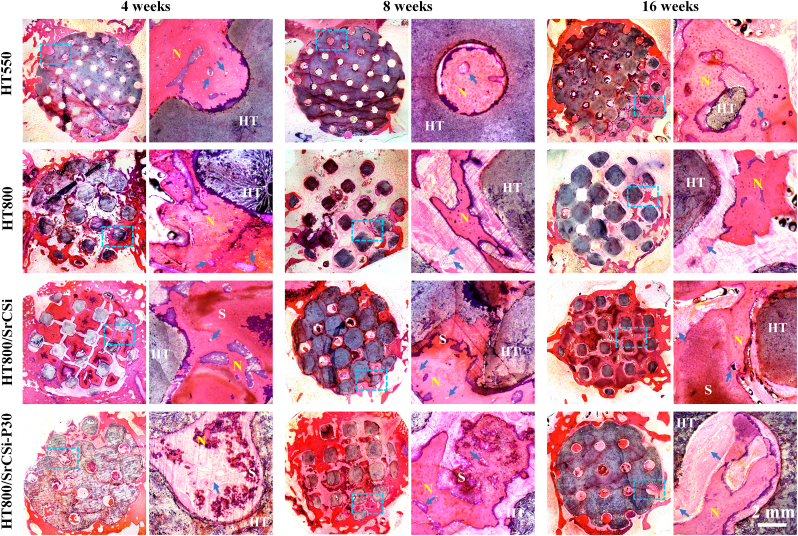
Histologic section analysis (H&E staining; 10 × , 40 × ) of specimens from 4 to 16 weeks postoperatively. N: neo-bone tissue; HT: hardystonite scaffold; S: SrCSi ceramic. Blue arrow: blood vessels. (For interpretation of the references to colour in this figure legend, the reader is referred to the Web version of this article.)

Histological staining of the specimens was performed to compare the differences between the four groups of HT-based scaffolds in promoting bone repair processes *in vivo*. HE staining results (Fig. 7) revealed a mild inflammatory reaction in all scaffolds at 4 weeks, with inflammatory cells visible at high magnification. At this time point, new bone tissue primarily occupied the peripheral macropores of bioceramic scaffolds, and the SrCSi filler caused internal macropores to be blocked, with a clear interface between new bone tissue and the SrCSi filler. After 16 weeks, the structures of all four groups remained intact, and the internal pores of the HT800/SrCSi–P30 group were filled in with neo-bone tissue completely. The SrCSi filler inside the HT800/SrCSi–P30 group was completely degraded, and the pores were filled with new bone tissue, at this time.

**Fig. 8:**
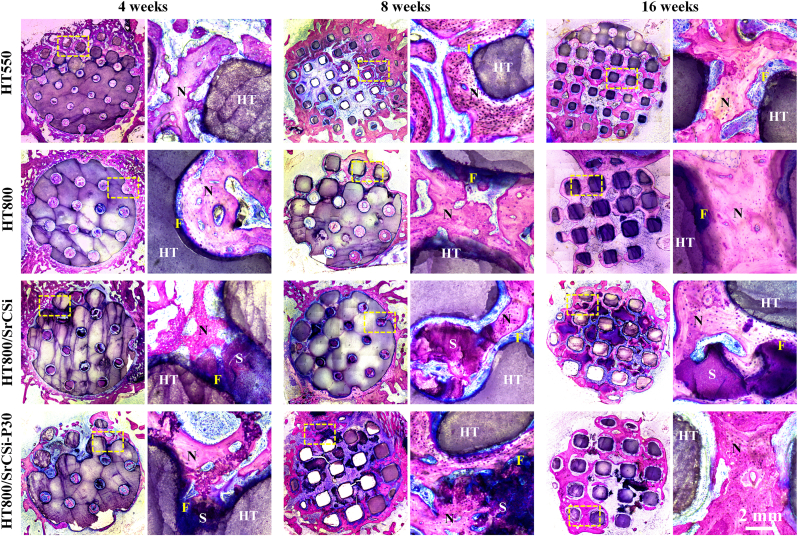
McNeal staining (10 × , 40 × ) of femoral samples in cross-section at 4, 8 and 16 weeks of implantation. N: neo-bone tissue; HT: hardystonite scaffold; S: SrCSi ceramic. F: Fibrous tissue.

McNeal-stained histological sections (Fig. 8), with unmineralized bone tissue stained blue and mineralized bone tissue stained red, showed numerous osteoid collagen deposits at the tissue-material interface in the SrCSi filling scaffold group at 4 weeks. In contrast, the HT550 group exhibited relatively rapid new bone growth, with a higher percentage of new bone area (8.59 ± 1.51 %). Over time, new bone tissue progressively infiltrated the scaffold, leading to a substantial increase in newly formed bone area. Notably, the HT800/SrCSi–P30 group exhibited progressively faster new bone growth with the degradation of SrCSi filler, with the highest relative bone area after 16 weeks (24.12 ± 1.75 %; Fig.S1).

### 3.8 *In vivo* phase conversion evaluation of scaffolds

**Fig. 9:**
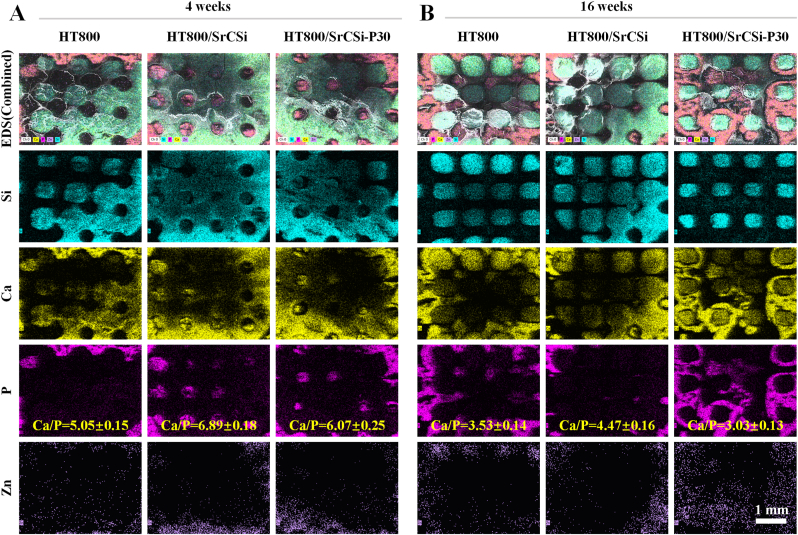
Phase transition analysis of scaffolds *in vivo*. SEM images and EDS mapping (25 × ) of Si, Ca, P, and Zn in the defect region after 4 weeks (A) and 16 weeks (B) of implantation. The different components overlap in the EDS profile.

SEM-EDS analysis was utilized to assess the phase transition process and element distribution in the bone defects at 4 and 16 weeks after surgery (Fig. 9). The EDS map revealed the neo-bone tissue, residual scaffold, and an intermediate transition zone. The residual scaffold area was rich in Ca, Zn, and Si, while the neo-bone tissue region exhibited higher Ca and P contents. As the implantation time extended to 16 weeks, the Si-rich zone inside the bioceramic composite scaffolds decreased, replaced by an apatite mineral layer rich in calcium and phosphorus. Especially in the HT800/SrCSi–P30 group, the entire interior of the scaffold was completely covered with new bone. Additionally, the Ca/P ratio (shown in the P mapping) decreased over time in all groups. Among them, the Ca/P of the HT800/SrCSi–P30 group decreased from 6.07 ± 0.25 at 4 weeks to 3.03 ± 0.13 at 16 weeks. This is possibly due to the continuous deposition of new bone and apatite-like layer during the implantation period.

## 4 Discussion

It is well known that space maintenance is a critical requirement for successful bone repair as it provides a 3D environment for new bone tissue growth [37]. While calcium phosphate-based (hybridized) porous scaffold biomaterials have gained recent attention, they often exhibit suboptimal bioreactivity *in vivo* and inadequate mechanical strength [38,39]. Conversely, traditional biodegradable materials may lack the strength required to support large bone defects effectively, thus failing to achieve desired osteogenic outcomes [[40], [41], [42]]. Consequently, there is a pressing need for the development of bone implants that combine mechanical strength and osteogenic activity to address clinical requirements.

DLP-based stereolithography allows precise control of pore geometry (including porosity, pore shape, and pore size) in 3D models at a low cost. It enables the fine-tuning of the internal structure and pore interconnectivity of scaffolds [[43], [44], [45]]. Zadpoor et al. have demonstrated that scaffold pore geometry significantly influences mechanical properties and tissue regeneration capabilities [46]. It is widely thought that for optimal nutrient exchange and regenerative tissue growth, bioceramic scaffolds should have pore sizes larger than 500 μm and porosities of at least 50 % [47,48]. Our previous research revealed that cylindrical pore scaffolds not only possess high compression and bending strength but also enhance the expression of osteogenesis-related genes [18,36]. Hence, in this study, we developed scaffolds with substantial macropore sizes and modified them based on 800-pore-size scaffolds to assess their osteogenic capabilities and mechanical characteristics (Scheme 1).

Observations, both macroscopic and microscopic, confirm that the printed scaffolds maintain their predefined structural features (Fig. 1, Fig. 2). For biphasic composite scaffolds, the internally interconnected porous structure is filled with SrCSi entirely. In fact, an ideal bone scaffold must provide temporary support with good mechanical properties and stability [21]. HT bioceramics, incorporating zinc into the Ca–Si oxide system, exhibit higher mechanical strength than pure HA and β-TCP bioceramics [34,49]. Moreover, HT bioceramics offer controlled biodegradation rates, making them suitable for stable porous scaffolds compared with pure silicate bioceramics [25,50]. In this context, all scaffolds in our study displayed excellent compressive strengths (>20 MPa). The strength (over 13 MPa) was maintained after 8 weeks of immersion in an aqueous medium (Fig. 3D), which significantly outperformed cancellous bone (2–6 MPa) [51,52]. Importantly, the four groups of scaffolds exhibited mild mechanical decay over time, indicating their ability to remain stable for extended periods. Interestingly, SrCSi-filling bioceramic scaffolds maintained relatively stable mechanical support during the immersion period due to the stable pore structure of the HT substrate in composite scaffolds, even as SrCSi rapidly degraded. In this regard, the pore structure and mechanical properties of the scaffolds gradually approached those of the HT800 group with increased immersion time.

In general, assessing the *in vitro* biodegradability of bioceramics often relies on measuring mass loss in a pH-buffered medium. When examining the mass decay curves, it was found that SrCSi exhibited a faster rate of bio-dissolution compared to the highly sparingly dissolvable HT (Fig. 3I). This observation was further supported by the ion release behavior (Fig. 3E-H). As expected, the HT800/SrCSi–P30 scaffolds exhibited more rapid rates of mass loss and ion release than the HT800/SrCSi, primarily attributed to their higher porosity and specific surface area, which accelerated ion dissolution from the porous bioceramic, favoring faster ion exchange between the biomaterials and the aqueous medium [53]. Previous studies have indicated that despite the regulation of repair and regeneration processes in natural tissues by various cytokines, signaling molecules, and the local immune microenvironment, bioceramic scaffolds with controlled biodegradability can also offer the necessary biological cues for tissue regeneration and the regulation of cellular behavior [[54], [55], [56]]. Biodegradable ceramics can induce a mild host immune response and stimulate osseointegration and bone regeneration by releasing bioactive ions [57].

**Scheme 2:**
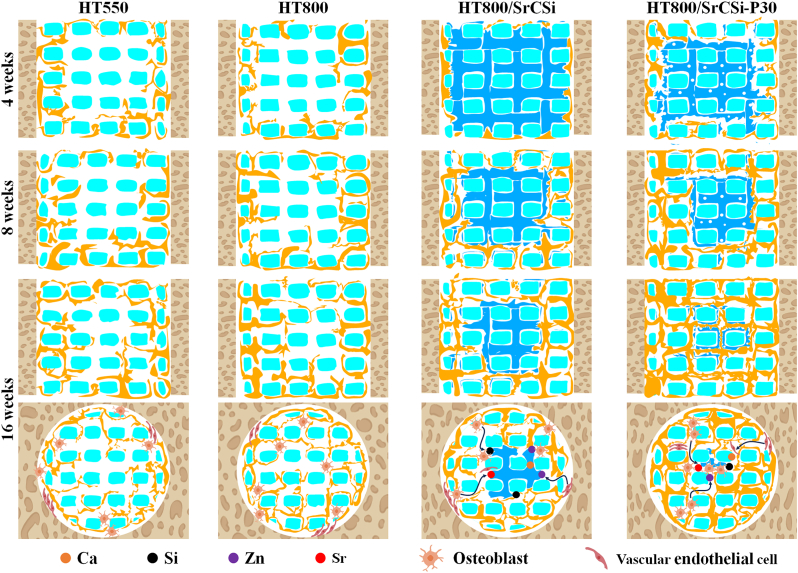
Schematic illustration of the biodegradation and new bone growth process of the four groups of scaffolds *in vivo*. Blue: HT scaffold; Green: SrCSi ceramic; Yellow: neo-bone tissue.

Bioceramic scaffolds need to exhibit appropriate degradation rates to provide the necessary bioactive ions for osteoblasts' value addition, differentiation, and bone regeneration. As bone primarily consists of calcium, variations in its content directly influence bone growth and development. A specific concentration of calcium supports the proliferation, differentiation, and mineralization of osteoblasts [58]. Numerous studies have demonstrated that silicon not only enhances osteoblast proliferation by regulating their differentiation cycle but also possesses a strong ability to induce hydroxyapatite formation, effectively stimulating osseointegration [59]. Lin et al. indicated that strontium doping at a certain concentration could effectively promote bone repair because Sr^2+^ not only increases the expression of osteoblast-related genes and enhances alkaline phosphatase (ALP) activity but also inhibits osteoclast differentiation by suppressing the expression of the nuclear factor kappa-B ligand-receptor activator in MSCs [30,60]. In this context, the results of our *in vitro* osteogenesis experiments also showed that the introduction of SrCSi components significantly increased the expression of osteogenesis-related proteins and genes in BMSCs (Fig. 4). We also evaluated the *in vivo* osteogenic effects of different porous structural scaffolds by μCT reconstruction (Fig. 6). During the early implantation period (4 weeks), the pure HT550 scaffolds exhibited a higher bone volume fraction and new bone ingrowth, which can be attributed to the insufficient biodegradation of SrCSi in the macropores of HT-based scaffolds, which hinders blood vessel and bone tissue ingrowth. As expected, the HT800/SrCSi–P30 group showed a significant improvement in bone regeneration and repair with prolonged implantation time, which was attributed to the continuous degradation of the SrCSi filler in the macroporous pore and the sustained release of biologically active ions, which not only provided sufficient space for new bone growth but also sustained induction of inward migration of osteoblasts and vascular endothelial cells (Scheme 2). Interestingly, as the SrCSi component degraded, the scaffolds gradually changed from micropores to open pore structures and finally formed large interconnected pore structures. Earlier research has suggested that the combination of interconnected micropores and macropores can yield a synergistic effect, promoting osteogenic cell migration [61]. Hossein et al. also proposed that the coexistence of macropores and micropores could lead to enhanced osteogenesis [62]. Therefore, it is reasonable to assume that the dynamic change of pore structure from micropore to macropore may be more beneficial for bone repair.

Moreover, improving bone regeneration and repair typically requires addressing issues related to anti-infection and immunomodulation [63]. Previous studies have demonstrated that HT bioceramic exhibits potential for pro-vascular growth and antibacterial properties [28]. Additionally, the release of Zn^2+^ can promote bone immunomodulation by inducing macrophage M_2_ polarization through the PI_3_K/Akt/mTOR pathway [64,65]. However, due to its limitations in terms of bioactivity and osteoinductive potential, our experiments addressed this deficiency by introducing strontium-doped CSi. The results of *in vitro* and *in vivo* osteogenic experiments demonstrated that the introduction of SrCSi significantly enhanced the bioactivity and osteogenic efficiency of HT-based scaffolds. In total, our findings show that the fast biodegradation rate of the internally filled bioceramic component has a significance on the bone tissue ingrowth in low-bioactivity and even bio-inert porous substrates, and such composite scaffold design is facile and versatile for developing some other advanced porous biomaterial systems such as biomedical metal and alloy scaffolds which may resolve some challenging clinical problems in load-bearing bone defect conditions.

## 5 Conclusion

To summarize, we successfully developed new bioceramic composite scaffolds with precise control over component distribution and systematically evaluated their mechanical and osteogenic performance. The SrCSi-filled HT-based composite scaffolds exhibited not only significant compressive strength and modulus but also demonstrated appreciable biological activity such as promoting the proliferation and differentiation of osteoblast stem cells and the expression of *Col1α1*, *Spp1*, and *Bmp2* genes. Notably, our *in vivo* bone repair results revealed that while the SrCSi filler may initially slow down new bone regeneration, it significantly enhanced bone tissue ingrowth with extended implantation time. Importantly, the high-porosity SrCSi filler group exhibited exceptionally high osteogenic capabilities compared to other scaffold conditions. It is believed that these new findings are valuable for the development of mechanically strong composite scaffolds that can provide the expected structural stability and biological performance by using the conventional suboptimal bioactive or bio-inert metallic and nonmetallic biomaterials for a wide range of bone repair and reconstruction applications.

## CRediT authorship contribution statement

**Xiaoyi Jiao:** Conceptualization, Investigation, Writing - original draft. **Fanghui Wu:** Conceptualization, Investigation, Writing - original draft. **Xusong Yue:** Data curation, Validation. **Jun Yang:** Data curation, Visualization. **Yan Zhang:** Data curation, Visualization. **Jiandi Qiu:** Software, Validation. **Xiurong Ke:** Software, Validation. **Xiaoliang Sun:** Supervision. **Liben Zhao:** Supervision. **Chuchu Xu:** Software, Validation. **Yifan Li:** Software, Validation. **Xianyan Yang:** Supervision. **Guojing Yang:** Writing - review & editing. **Zhongru Gou:** Project administration, Writing - review & editing. **Lei Zhang:** Project administration, Writing - review & editing.

## Declaration of competing interest

The authors declare that they have no known competing financial interests or personal relationships that could have appeared to influence the work reported in this paper.

## Figures and Tables

**Figure:**
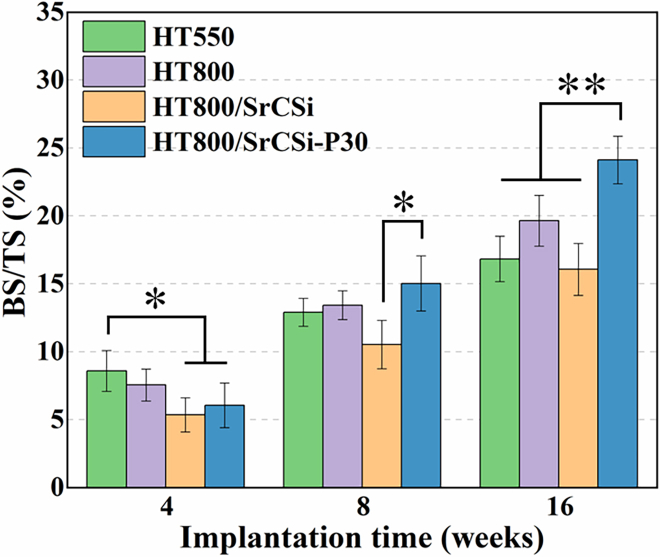
Fig. S1 Percentage of new bone area in each group of sections at different implantation periods. (*p＜0.05, **p＜0.01).

## Data Availability

Data will be made available on request.
