# Supplementary material for: New insight into biodegradable macropore filler on tuning mechanical properties and bone tissue ingrowth in sparingly dissolvable bioceramic scaffolds
Source: Mater Today Bio. 2023 Dec 28;24:100936. doi: 10.1016/j.mtbio.2023.100936 (PMC10792586; doi:10.1016/j.mtbio.2023.100936)
Supplement: Multimedia component 1 [file mmc1.docx]

**Table S1**. Primers sequences used in qRT-PCR

| Gene | Primer sequences |
| --- | --- |
| *Col1α1* | F: 5’- TGTTGGTCCTGCTGGCAAGAATG -3’ |
|  | R: 5’- GTCACCTTGTTCGCCTGTCTCAC -3’ |
| *Spp1* | F: 5’- GACGATGATGACGACGACGATGAC -3’ |
|  | R: 5’- GTGTGCTGGCAGTGAAGGACTC -3’ |
| *Bmp2* | F: 5’- GGAGGAGGTGAAGAAAGGCAACAG -3’ |
|  | R: 5’- CCGCAGTCCGTCTAAGAAGCAC -3’ |
| *Gapdh* | F: 5’- ACGGCAAGTTCAACGGCACAG -3’ |
|  | R: 5’- CGACATACTCAGCACCAGCATCAC -3’ |
